# A-MYB/TCFL5 regulatory architecture ensures the production of pachytene piRNAs in placental mammals

**DOI:** 10.1261/rna.079472.122

**Published:** 2023-01

**Authors:** Tiangxiong Yu, Adriano Biasini, Katharine Cecchini, Martin Säflund, Haiwei Mou, Amena Arif, Atiyeh Eghbali, Dirk G. de Rooij, Zhiping Weng, Phillip D. Zamore, Deniz M. Özata

**Affiliations:** 1 Program in Bioinformatics and Integrative Biology, University of Massachusetts Medical School, Worcester, Massachusetts 01605, USA; 2 RNA Therapeutics Institute and Howard Hughes Medical Institute, University of Massachusetts Medical School, Worcester, Massachusetts 01605, USA; 3 Department of Molecular Biosciences, The Wenner-Gren Institute, Stockholm University, S-106 91 Stockholm, Sweden; 4 Cold Spring Harbor Laboratory, Cold Spring Harbor, New York 11724, USA; 5 Reproductive Biology Group, Division of Developmental Biology, Department of Biology, Faculty of Science, Utrecht University, Utrecht 3584, the Netherlands

**Keywords:** TCFL5, A-MYB, pachytene piRNAs, spermatogenesis

## Abstract

In male mice, the transcription factor A-MYB initiates the transcription of pachytene piRNA genes during meiosis. Here, we report that A-MYB activates the transcription factor *Tcfl5* produced in pachytene spermatocytes. Subsequently, A-MYB and TCFL5 reciprocally reinforce their own transcription to establish a positive feedback circuit that triggers pachytene piRNA production. TCFL5 regulates the expression of genes required for piRNA maturation and promotes transcription of evolutionarily young pachytene piRNA genes, whereas A-MYB activates the transcription of older pachytene piRNA genes. Intriguingly, pachytene piRNAs from TCFL5-dependent young loci initiate the production of piRNAs from A-MYB-dependent older loci, ensuring the self-propagation of pachytene piRNAs. A-MYB and TCFL5 act via a set of incoherent feedforward loops that drive regulation of gene expression by pachytene piRNAs during spermatogenesis. This regulatory architecture is conserved in rhesus macaque, suggesting that it was present in the last common ancestor of placental mammals.

## INTRODUCTION

In human and mouse testes, ∼100 genes—first expressed at the pachytene stage of meiosis I—make noncoding transcripts that are processed into 26–30 nt pachytene PIWI-interacting RNAs (pachytene piRNAs) (Li et al. 2013; Özata et al. 2020). Found only in placental mammals, pachytene piRNAs are diverse—comprising ∼1 million distinct species—and plentiful—rivaling ribosomal RNA abundance (Li et al. 2013; Gainetdinov et al. 2018; Özata et al. 2020). Pachytene piRNAs bind the PIWI proteins PIWIL1 and PIWIL2, generating RNA–protein complexes that can repress transcripts during male gametogenesis (Aravin et al. 2006; Girard et al. 2006; Grivna et al. 2006; Lau et al. 2006; Goh et al. 2015; Han et al. 2015; Homolka et al. 2015; Mohn et al. 2015; Özata et al. 2020; Wu et al. 2020; Choi et al. 2021). Successful spermiogenesis requires pachytene piRNAs, yet their sequences are poorly conserved and rapidly diverging even among modern humans (Özata et al. 2020; Wu et al. 2020; Choi et al. 2021).

The transcription factor A-MYB binds the promoters of more than half of pachytene piRNA-producing genes (“pachytene piRNA genes”) in humans and mice, and *A*-*Myb* mutant mice make fewer pachytene piRNAs than wild-type (Li et al. 2013; Özata et al. 2020). Although *A-Myb*-mutant germ cells fail to express meiotic genes and arrest early in meiosis I (Bolcun-Filas et al. 2011), human and mouse testes with little or no A-MYB still make some pachytene piRNAs (Özata et al. 2020); and the promoters of half of human and macaque piRNA-producing genes are not bound by A-MYB (Özata et al. 2020), suggesting that one or more additional transcription factors act downstream or in parallel to A-MYB to initiate piRNA production.

Here, we report that A-MYB initiates transcription of *Tcfl5* early in meiosis I. Subsequently, TCFL5 binds both its own promoter and that of *A*-*Myb*, establishing a mutually reinforcing positive feedback loop. Whereas A-MYB generally initiates the production of pachytene piRNAs from evolutionarily older piRNA precursor loci, transcription of younger pachytene piRNA genes is more dependent on TCFL5. Like A-MYB, TCFL5 promotes transcription of both piRNA precursors and mRNAs encoding piRNA biogenesis proteins. The A-MYB/TCFL5 positive feedback loop allows each protein to initiate interlocking coherent feedforward loops dedicated to piRNA production. Together, A-MYB and TCFL5 regulate the transcription of both pachytene piRNAs and their target transcripts, establishing an incoherent feedforward loop during spermatogenesis. This transcriptional architecture is evolutionarily conserved in the old-world monkey rhesus macaque, suggesting it predates the divergence of rodents and primates. Our findings suggest that this transcriptional circuit likely coevolved with pachytene piRNAs themselves.

## RESULTS

### A-MYB is expressed before TCFL5 during male meiosis I

**FIGURE 1.**
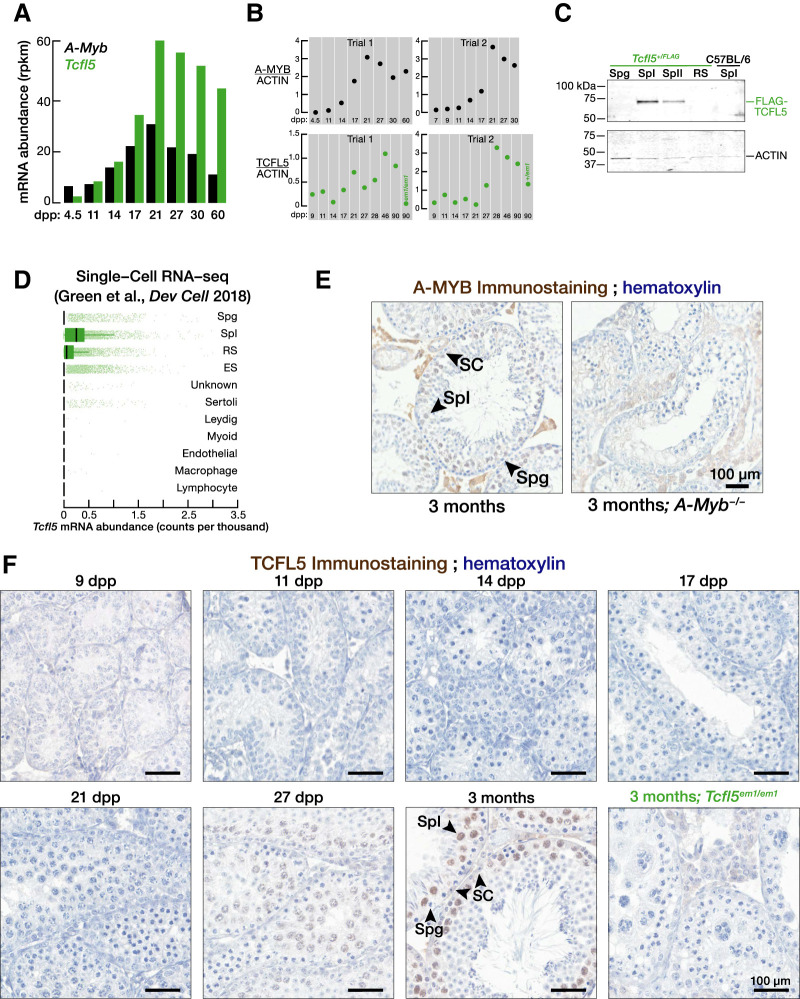
TCFL5 is expressed in pachytene spermatocytes during male meiosis I. (*A*,*B*) *A*-*Myb* and *Tcfl5* mRNA (A) and protein abundance (*B*) at distinct developmental stages of mouse spermatogenesis. See Supplemental Figure S1A for uncropped western blot images. (*C*) Abundance of FLAG-TCFL5 protein in FACS-purified germ cell from *Tcfl5*^*+/FLAG*^ mouse. Each lane contained protein from 75,000 germ cells. (*D*) mRNA abundance of *Tcfl5* mRNA measured by single-cell sequencing by Green et al. (2018). (*E*) Immunohistochemical detection of A-MYB in adult testis section. *A*-*Myb*^−*/*−^ testis sections serve as antibody specificity controls. (*F*) Immunohistochemical detection of TCFL5 in testis sections from staged mice. *Tcfl5*^*em1/em1*^ testis sections serve as antibody specificity controls. (Spg) Spermatogonia, (SpI) primary spermatocytes, (SpII) secondary spermatocytes, (RS) round spermatids, (ES) elongating spermatids, (SC) Sertoli cells.

Consistent with the testis-specific expression of human *Tcfl5* (Maruyama et al. 1998), two recent studies revealed TCFL5 is first expressed in mouse primary spermatocytes (Galán-Martínez et al. 2022; Xu et al. 2022). We used staged mouse testes (Nebel et al. 1961) to measure the abundance of *Tcfl5* mRNA and protein across spermatogenesis. At 11 d post-partum (dpp), meiosis proceeds no further than the zygotene stage; early pachytene spermatocytes appear at 14 dpp; and mid- and late-pachytene spermatocytes are present by 17 dpp. *A*-*Myb* mRNA and protein were first detected at 14 dpp. In contrast, *Tcfl5* mRNA was present at 14 dpp, but TCFL5 protein was not detected until after 17 dpp (Fig. 1A,B; Supplemental Fig. S1A).

To directly test whether TCFL5 is expressed in primary spermatocytes, we purified germ cells from *Tcfl5*^*+/em2(flag)Pdz*^ (henceforth, *Tcfl5*^*+/FLAG*^) mice in which the endogenous TCFL5 protein is tagged with 3XFLAG peptide at its amino terminus (Supplemental Fig. S1B) and performed western blotting using anti-FLAG antibody. FLAG-TCFL5 was not detected in purified spermatogonia; was expressed abundantly in primary spermatocytes; and was present at a lower level in secondary spermatocytes (Fig. 1C; Supplemental Fig. S1C). Reinforcing the view that *Tcfl5* is expressed mostly in primary spermatocytes, we reanalyzed publicly available single-cell RNA-sequencing data (Green et al. 2018): *Tcfl5* mRNA abundance was highest in primary spermatocytes compared to spermatogonia, spermatids, and testicular somatic cells (Fig. 1D). Moreover, consistent with the primary spermatocyte-specific expression of A-MYB (Fig. 1E; Tang and Goldberg 2012), immunostaining of TCFL5 in testis sections from staged mouse detected the TCFL5 in the nuclei of primary spermatocytes, but not in those of spermatogonia or Sertoli cells (Fig. 1F). In contrast to previous observations (Xu et al. 2022), we detected a single, well-resolved TCFL5 band by western blotting. A single TCFL5 protein isoform is consistent with publicly available chromatin immunoprecipitation followed by sequencing (ChIP-seq) of histone H3 trimethylated on lysine 4 (H3K4me3) (Maezawa et al. 2018) and transposase-accessible chromatin with sequencing (ATAC-seq) data from purified primary spermatocytes (Maezawa et al. 2018), which detect a single prominent peak of H3K4me3 or ATAC-seq around the transcription start site of *Tcfl5* gene (Supplemental Fig. S1D).

**FIGURE 2.**
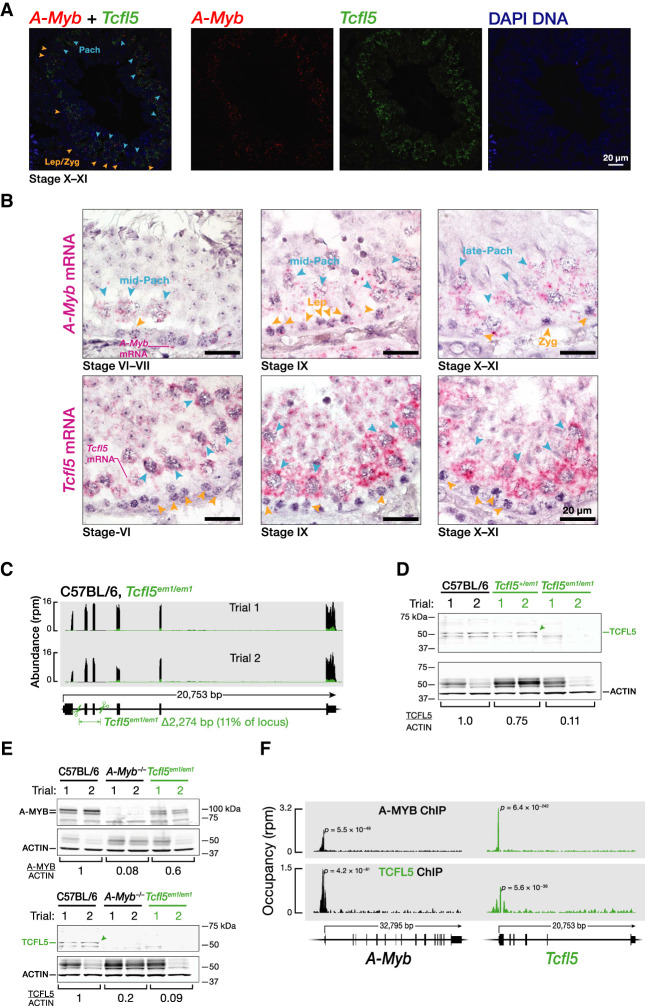
*A*-*Myb* expression precedes that of *Tcfl5*. (*A*,*B*) Temporal *A*-*Myb* and *Tcfl5* mRNA expression in a seminiferous tubule section from adult mouse testis was detected. Two-color RNA fluorescent in situ hybridization (RNA-FISH) (*A*). Single-color RNA-FISH (*B*). (*C*) Strategy to generate *Tcfl5* knockout mice using a single guide RNA (sgRNA) and Cas9. Scissors indicate sites targeted by sgRNAs designed to delete *Tcfl5* exons 2 and 3. RNA-seq was used to measure the abundance of *Tcfl5* mRNA in adult testes. (*D*) Abundance of TCFL5 protein in *Tcfl5*^*em1/em1*^ homozygous and *Tcfl5*^*+/em1*^ heterozygous mutant testes, relative to C57BL/6 wild-type testes. ACTIN serves as a loading control. Each lane contained 50 µg testis protein. (*E*) Abundance of A-MYB and TCFL5 proteins in *A*-*Myb*^−*/*−^ and *Tcfl5*^*em1/em1*^ homozygous mutant mouse testes. ACTIN serves as a loading control. Each lane contained 50 µg testis protein. (*F*) A-MYB and TCFL5 ChIP-seq peaks at the promoters of the *A*-*Myb* and *Tcfl5* genes.

*A*-*Myb* expression precedes that of *Tcfl5* (Fig. 2A): dual-color RNA fluorescence in situ hybridization (RNA-FISH) of adult testis sections revealed that *A*-*Myb* mRNA first appeared in the nuclei of leptotene/zygotene cells as one or two brightly staining foci, likely corresponding to its genomic sites of transcription (Supplemental Fig. S2A). *Tcfl5* mRNA was not detected in leptotene/zygotene cells, and first appeared in the nuclei and cytoplasm of pachytene cells. Single-color RNA-FISH of multiple seminiferous tubules at different stages confirmed that *A*-*Myb* mRNA first appears in preleptotene, leptotene, and zygotene cells, whereas *Tcfl5* first appears in mid- and late-pachytene cells (Fig. 2B).

### A-MYB initiates a reciprocal positive feedback loop between TCFL5 and A-MYB

*Tcfl5* mRNA was not detectable by RNA-FISH in *A*-*Myb*^*repro9/repro9*^ (henceforth, *A*-*Myb*^−*/*−^) mutant testis (Supplemental Fig. S2B). We used a pair of single-guide RNAs (sgRNAs) to generate a *Tcfl5* knockout by deleting *Tcfl5* exons 2 and 3 (2274 bp) (Fig. 2C,D; Supplemental Fig. S2C). *A*-*Myb* mRNA was readily detected in *Tcfl5*^*em1Pdz/em1Pdz*^ (henceforth, *Tcfl5*^*em1/em1*^) (Supplemental Fig. S2B). A-MYB protein was less abundant in *Tcfl5*^*em1/em1*^ than in C57BL/6 control testes (Fig. 2E; Supplemental Fig. S2D). In contrast, no TCFL5 protein was detected in either *A*-*Myb*^−*/*−^ or *Tcfl5*^*em1/em1*^ mutants (Fig. 2E; Supplemental Fig. S2D). These data suggest that A-MYB initiates *Tcfl5* expression, while TCFL5 acts to increase the steady-state abundance of A-MYB.

To determine whether A-MYB regulates *Tcfl5* directly or indirectly, we performed ChIP-seq of adult mouse testes. A prominent A-MYB peak was located at the first nucleotide of the *Tcfl5* transcription start site (Fig. 2F). TCFL5 ChIP-seq also revealed a significant TCFL5 peak within the promoters of both *A*-*Myb* and *Tcfl5* itself (Fig. 2F). A-MYB and TCFL5 cleavage under targets & release using nuclease (CUT&RUN; Skene and Henikoff 2017) of FACS-purified primary spermatocytes confirmed these observations (Supplemental Fig. S2E). We conclude that A-MYB promotes its own transcription and directly initiates transcription of *Tcfl5*; TCFL5 responds by reinforcing its own transcription and that of A-MYB via positive transcriptional feedback.

### TCFL5 regulates the expression of piRNA pathway genes

At the onset of the pachynema in mice and humans, A-MYB activates transcription of genes producing pachytene piRNA precursors and genes encoding piRNA biogenesis proteins, thereby generating a feedforward loop that drives pachytene piRNA production (Li et al. 2013; Özata et al. 2020). Given the expression pattern of TCFL5 and A-MYB during mouse spermatogenesis and the previously identified requirement for *A-Myb* in pachytene piRNA production, we sought to test if TCFL5 also plays a role in pachytene piRNA production.

**FIGURE 3.**
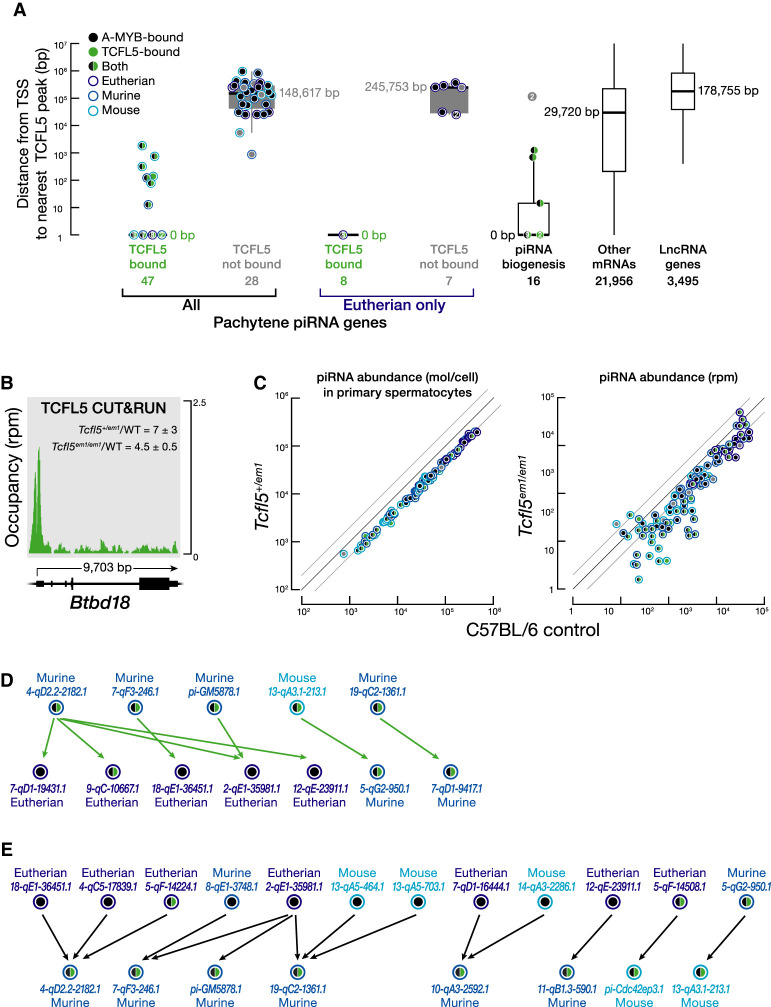
Younger pachytene piRNA genes whose transcription is activated by TCFL5 produce piRNAs that initiate piRNA production in evolutionarily older pachytene piRNA genes. (*A*) The distance from the nearest TCFL5 peak to the transcription start sites (TSS) of pachytene piRNA genes, genes encoding piRNA biogenesis proteins, and other protein-coding or noncoding genes obtained from CUT&RUN of FACS-purified primary spermatocytes. *Horizontal* lines: median; whiskers: maximum and minimum values, excluding outliers. Each dot represents distance (mean of two trials) from the nearest TCFL5 peak to the transcription start site of individual genes. Measurements with the same values are indicated by a single marker indicating the number of individual data points. *Gpat2*, which is required for piRNA biogenesis in flies (Vagin et al. 2013) and in cultured mouse germline stem cells (Shiromoto et al. 2013), was not included in this analysis, because its participation in the piRNA pathway in vivo in mice remains to be established. (*B*) TCFL5 CUT&RUN peak at the promoter of *Btbd18*. WT denotes C57BL/6. (*C*) Steady-state abundance of pachytene piRNAs in primary spermatocytes (*left*) or whole testes (*right*) for *Tcfl5*^*+/em1*^ heterozygotes compared to C57BL/6 controls. Each marker represents a pachytene piRNA gene. (*D*,*E*) Pachytene piRNA-directed cleavage sites in pachytene piRNA precursor transcripts. In *D*, each arrow points from the piRNAs derived from a TCFL5-dependent pachytene piRNA gene toward the corresponding cleavage site in a pachytene piRNA precursor transcript whose transcription is activated by A-MYB. TCFL5-dependent genes were defined as those whose promoters were occupied by TCFL5 and whose precursor and piRNA abundance in *Tcfl5*^*em1/em1*^ mutant mice were less than those in C57BL/6. In *E*, each arrow points from the piRNAs derived from one of 12 A-MYB-dependent pachytene piRNA genes toward genes for which the abundance of pachytene piRNA precursor transcripts was reduced less than twofold in *Tcfl5*^*em1/em1*^ mutant mice. A-MYB-dependent genes were defined as those whose promoters were occupied by A-MYB and whose expression was essentially unchanged in *Tcfl5*^*em1/em1*^ mutant mice.

In FACS-purified primary spermatocytes, the promoters of 14 piRNA biogenesis genes featured a TCFL5 peak near their transcription start sites (χ^2^ test ρ = <2.7 × 10^−9^; median distance = 0 bp) (Fig. 3A; Supplemental Fig. S3A; Supplemental Table S1A). The steady-state mRNA abundance of four of the TCFL5-bound piRNA biogenesis genes—*Henmt1*, *Mael, Pld6*, and *Tdrd6—*was reduced greater than or equal to twofold in *Tcfl5*^*em1/em1*^ and *Tcfl5*^*+/em1*^ testes (Supplemental Fig. S3A,B; Supplemental Table S1A). Among the remaining ten piRNA biogenesis genes—*Piwil1*, *Piwil2*, *Ddx39*, *Tdrd12*, *Tdrd5*, *Tdrd9*, *Tdrd1*, *Mov10l1*, *Fkbp6*, and *Ddx4*—the mRNA abundance of five decreased modestly in *Tcfl5*^*+/em1*^ (Supplemental Fig. S3A; Supplemental Table S1A). The promoters of the nine genes were all bound by A-MYB, suggesting that their transcription is either driven entirely by A-MYB or that A-MYB largely compensates in *Tcfl5* mutant mice. We conclude that in parallel to A-MYB, TCFL5 also establishes a feedforward loop that powers pachytene piRNA production.

### TCFL5 activates evolutionarily younger pachytene piRNA genes

Our data suggest that TCFL5 activates transcription of almost half of pachytene piRNA genes during male meiosis I. CUT&RUN of FACS-purified primary spermatocytes identified TCFL5 peaks within 500 bp of the transcription start sites of 47 of the 100 mouse pachytene piRNA genes (median distance = 0 bp), whereas 91 were bound by A-MYB (median distance = 0 bp) (Fig. 3A; Supplemental Table S1B).

Pachytene piRNA genes with evolutionary conservation of genomic location (synteny) have first exons longer than 10 kbp and a low level of CG dinucleotides, and their promoters and gene bodies are bound by the transcription elongation factor BTBD18 and marked by histone lysine acylation, including acetylation, butyrylation, and crotonylation (Zhou et al. 2017; Yu et al. 2021). BTBD18 encodes a transcriptional elongation factor that has been proposed to facilitate the production of piRNA precursors from evolutionarily older pachytene piRNA genes (Yu et al. 2021), and precursor transcript and mature piRNA abundance from these loci declines significantly in *Btbd18* mutant testes (Zhou et al. 2017). Although TCFL5 binds the *Btbd18* promoter (distance from transcription start site to the nearest TCFL5 peak = 0 bp), *Btbd18* mRNA increased in both *Tcfl5*^*em1/em1*^ mutant testes (mean increase = 4.5 ± 0.5 fold) and *Tcfl5*^*+/em1*^ primary spermatocytes (mean increase = 7 ± threefold) compared to wild-type (Fig. 3B). We do not yet know whether TCFL5 serves to repress BTBD18 expression. Increased BTBD18 levels in mutants may help explain why loss of TCFL5 has little effect on the transcription of many evolutionary older pachytene piRNA genes.

A-MYB occupancy was greatest at the promoters of pachytene piRNA genes with low CG dinucleotide content (Spearman's ρ = −0.35; Supplemental Fig. S4A), long first exons (ρ = 0.28), high histone lysine acylation (ρ = 0.38–0.63) and BTBD18 occupancy (ρ = 0.44; Supplemental Fig. S4A). High A-MYB and low TCFL5 promoter occupancy was also associated with pachytene piRNA genes producing abundant piRNAs (Supplemental Fig. S4B). In contrast, TCFL5 occupancy was correlated with high CG dinucleotide promoter sequence (Spearman's ρ = 0.67; Supplemental Fig. S4A), a feature more likely to be found in evolutionarily younger pachytene piRNA genes, which typically produce fewer piRNAs (Yu et al. 2021).

Given that pachytene piRNA genes are marked by histone lysine acylation, it is striking that TCFL5 binds the promoters of seven genes encoding histone acyl transferases (for review, see Sabari et al. 2017). Of these seven genes, four were also bound by A-MYB (Supplemental Fig. S4C). Histone acyl transferases use acetyl-, butyryl-, or crotonyl-CoA as acyl donors. Histone crotonylation, which promotes gene expression, is regulated by the intracellular concentration of crotonyl-CoA (Sabari et al. 2015). *Acss2* encodes a short chain acyl-CoA synthetase (Luong et al. 2000) that uses ATP to synthesize acetyl- and crotonyl-CoA. TCFL5 binds the *Acss2* promoter (Supplemental Fig. S4D).

Our data suggest that pachytene piRNA genes with high A-MYB occupancy are more likely to have conserved synteny than those with high TCFL5 and low A-MYB occupancy (Supplemental Table S1B). Mouse pachytene piRNA genes were generally present at syntenic sites in other placental mammals (eutheria) when their promoters had high A-MYB occupancy (median = 3.8 RPM, 95% confidence interval [CI] = 2.8–4.7) compared to loci found only in murine rodents (median = 2.6 RPM, 95% CI = 2.1–3.2) or only in mouse (median = 2.3 RPM, 95% CI = 1.6–3.2) (Supplemental Fig. S4E; Supplemental Table S1B). Conversely, TCFL5 promoter occupancy was higher for murine (median = 1.0 RPM, 95% CI = 0.6–2.3) and mouse pachytene piRNA genes (median = 1.3 RPM, 95% CI = 0.4–2.1) than at eutherian loci (median = 0.8 RPM, 95% CI = 0.5–1.1). Finally, the promoters of 20 of the 21 eutherian pachytene piRNA genes were bound by A-MYB, but only eight were occupied by TCFL5 (Fig. 3A). Our finding that A-MYB activates transcription of older pachytene piRNA genes, whereas TCFL5 tends to activate younger loci, suggests that acquisition of a TCFL5-binding site may facilitate the evolution of new pachytene piRNA genes.

### *Tcfl5* mutants have fewer pachytene piRNAs

For the majority of pachytene piRNA genes, piRNA abundance in *Tcfl5*^*em1/em1*^ testes (77 of 100 genes) and in *Tcfl5*^*+/em1*^ primary spermatocytes (74 of 100 genes) was less than half that in C57BL/6 controls (Fig. 3C). The affected pachytene piRNA genes include loci with either or both A-MYB or TCFL5 bound to their promoters, as well as five bound by neither transcription factor. The loss of pachytene piRNAs in *Tcfl5* mutants likely reflects the combined effects of reduced transcriptional activation by TCFL5 and A-MYB and reduced expression of pachytene piRNA biogenesis proteins. Loss of initiator piRNAs may also contribute to reduced piRNA levels.

piRNA production begins when a PIWI protein, loaded with an initiator piRNA, cleaves a complementary piRNA precursor to generate a 5′ monophosphorylated pre-pre-piRNA. Binding of a second PIWI protein to the 5′ end of the pre-pre-piRNA allows it to be further processed into a PIWI-loaded responder piRNA that can initiate additional piRNA production at fully or partially complementary sites (Brennecke et al. 2007; Gunawardane et al. 2007; Han et al. 2015; Mohn et al. 2015; Gainetdinov et al. 2018; Ozata et al. 2019; Wu et al. 2020). Do piRNAs from TCFL5-activated pachytene piRNA genes initiate production of piRNAs from precursors whose transcription depends solely on A-MYB?

To test the idea that production of A-MYB-dependent piRNAs requires initiation by piRNAs from loci whose transcription is activated by TCFL5, we identified A-MYB-dependent piRNA precursor transcripts whose abundance declined less than twofold in *Tcfl5*^*em1/em1*^ but whose piRNA abundance declined twofold or more, suggesting a defect in piRNA biogenesis rather than transcriptional activation. Among these, we searched for piRNAs from TCFL5-dependent pachytene piRNA genes capable of directing cleavage of precursor transcripts from other A-MYB-dependent pachytene piRNA genes. Candidate piRNAs were required (i) to have complete seed complementarity (g2–g7) to the A-MYB-dependent precursor RNA; (ii) to form at least ten additional base pairs within the region g8–g21 with that precursor; and (iii) be able to generate the 5′ end of a piRNA by PIWI-protein-catalyzed precursor RNA cleavage. The 5′ ends of 29 piRNAs from five different TCFL5-dependent pachytene piRNA precursors are predicted to target seven A-MYB-dependent pachytene piRNA precursors. Four of the five TCFL5-dependent pachytene piRNA genes are confined to murine rodents and one was found only in mouse; none are conserved among eutheria generally. In contrast, five of the seven pachytene piRNA genes targeted by piRNAs from TCFL5-dependent pachytene piRNA genes are present at syntenic sites in other eutherian species; the remaining two are found among murine rodents (Fig. 3D; Supplemental Table S2; Fisher's exact test *P* = 0.028 between the two sets of pachytene piRNA genes). Conversely, 62 piRNAs from 12 A-MYB-dependent pachytene piRNA genes are predicted to target the precursors from eight TCFL5-dependent pachytene piRNA genes. Seven of the 12 A-MYB-dependent pachytene piRNA genes are present at syntenic sites in other eutheria, two are found among murine rodents, while three were found only in mouse. Among the eight TCFL5-dependent pachytene piRNA genes targeted by these 12 loci, none were present in nonmurine species (Fig. 3E; Supplemental Table S2; Fisher's exact test *P* = 0.037 between the 12 A-MYB-dependent and the 8 TCFL5-dependent genes). These data suggest that pachytene piRNAs from TCFL5-dependent young loci initiate the processing of pachytene piRNA precursors from older loci that are generally controlled by A-MYB. Similarly, piRNAs from older pachytene piRNA genes initiate the production of piRNAs from the precursors of young loci controlled by TCFL5. Together, A-MYB and TCFL5 ensure the production of pachytene piRNAs during male meiosis I.

### Pachytene piRNA-driven gene regulation is part of an incoherent feedforward loop established by the A-MYB/TCFL5 regulatory architecture

Loss of *pi6* piRNAs results in compromised sperm function*—*attributable to the increased abundance of six mRNAs directly targeted by *pi6* piRNAs (Wu et al. 2020). Intriguingly, although *pi6* pachytene piRNAs begin to accumulate in pachytene spermatocytes (Li et al. 2013; Gainetdinov et al. 2018; Özata et al. 2020; Wu et al. 2020), the increase in the abundance of the six mRNAs is delayed until secondary spermatocytes and subsequent stages (Wu et al. 2020). The initiation of the expression of *pi6*—a locus with conserved synteny among eutherians—by A-MYB (Fig. 3A; Supplemental Fig. S5A) and the delay in the increase in abundance of target mRNAs until later stages of spermatogenesis in *pi6* mutants (Wu et al. 2020) suggest that pachytene piRNAs do not completely repress their targets until their targets reach a specific steady-state threshold, a signature feature of incoherent feedforward loops (Alon 2007). Could then A-MYB/TCFL5 regulatory architecture amplify the production of pachytene piRNA targets in parallel to pachytene piRNA genes themselves? Our data identified TCFL5 peaks within 500 bp of the transcription start sites of five of the six genes whose mRNAs are directly targeted by *pi6* piRNAs, significantly more than detected for protein-coding genes generally (χ^2^ test *P* = <1.9 × 10^−3^; Supplemental Fig. S5B). Among those six genes, four were also bound by A-MYB (χ^2^ test *P* = <3.8 × 10^−6^; Supplemental Fig. S5B).

Mice lacking the *pi18* pachytene piRNA gene are sterile, and *pi18* piRNAs directly target the *Golga2* mRNA, whose steady-state abundance increases in *pi18* mutant mice (Choi et al. 2021). Our data revealed that the promoter of the *pi18* pachytene piRNA gene was bound by A-MYB and that the promoter of *Golga2* featured both A-MYB and TCFL5 peaks near its transcription start site (Fig. 3A; Supplemental Fig. S5A,C). Together, these data suggest that A-MYB and TCFL5 drive the production of both pachytene piRNAs and their targets, generating an incoherent feedforward loop that generates a pulse of expression of piRNA target mRNAs that is subsequently extinguished by piRNA-directed, PIWI-protein-catalyzed cleavage of the same mRNAs.

### The regulatory architecture that drives pachytene piRNA production in rodents is conserved in rhesus macaque

In both rodents and primates, A-MYB activation of piRNA pathway proteins and pachytene piRNA genes creates a coherent feedforward loop that ensures the robust accumulation of pachytene piRNAs (Li et al. 2013; Özata et al. 2020). In mouse, TCFL5 similarly organizes a coherent feedforward loop that activates the production of pachytene piRNAs.

**FIGURE 4.**
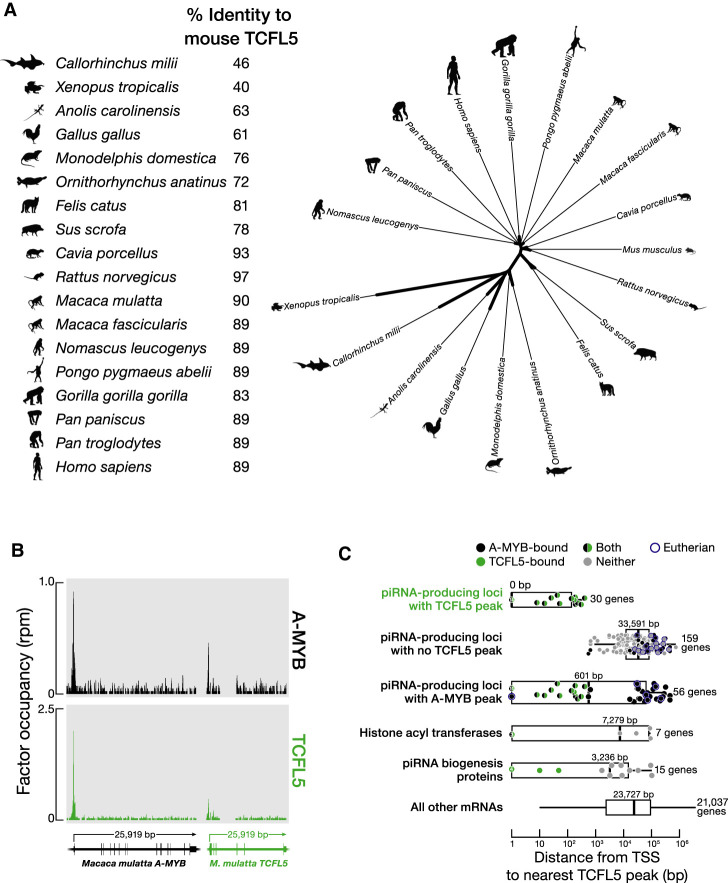
The role of TCFL5 in piRNA production is conserved in rhesus macaque. (*A, left*) Percent identity of mouse TCFL5 to TCFL5 in other mammals. (*Right*) Unrooted tree of TCFL5 protein sequences, aligned using Clustal Omega. The unrooted tree was constructed using Randomized Axelerated Maximum Likelihood with default parameters and visualized in the Interactive Tree Of Life. (*B*) A-MYB and TCFL5 ChIP-seq peaks at the promoters of the rhesus macaque *A*-*MYB* and *TCFL5* genes. (*C*) The distance from the nearest TCFL5 ChIP-seq peak to the transcription start site (TSS) for rhesus genes separated by class. *Vertical* lines: median; whiskers: maximum and minimum values, excluding outliers. A single marker containing the number of data points marks multiple identical values.

In addition to placental mammals, TCFL5 orthologs are found in the genomes of marsupials, monotremes, birds, reptiles, amphibians, and fishes (Fig. 4A). Thus, TCFL5 may participate in spermatogenesis across vertebrate species. Moreover, the A-MYB/TCFL5 regulatory architecture—reciprocal positive feedback circuits that reinforce *A*-*Myb* and *Tcfl5* transcription, sitting atop parallel A-MYB and TCFL5 coherent feedforward loops that drive pachytene piRNA production—is conserved in rhesus macaque (Fig. 4B,C; Supplemental Table S3). ChIP-seq of TCFL5 from adult rhesus testis identified 17,959 genomic regions with significant TCFL5 peaks (FDR < 0.05), comprising 4048 annotated genes. The TCFL5-bound genes include six of the 15 rhesus orthologs of known piRNA biogenesis proteins and 30 of the 189 rhesus piRNA genes (Özata et al. 2020) (median distance from transcription start site to nearest TCFL5 peak = 0 bp) (Fig. 4C). Together, these data suggest that the A-MYB/TCFL5 transcriptional architecture was present in the last common ancestor of rodents and primates.

## DISCUSSION

**FIGURE 5.**
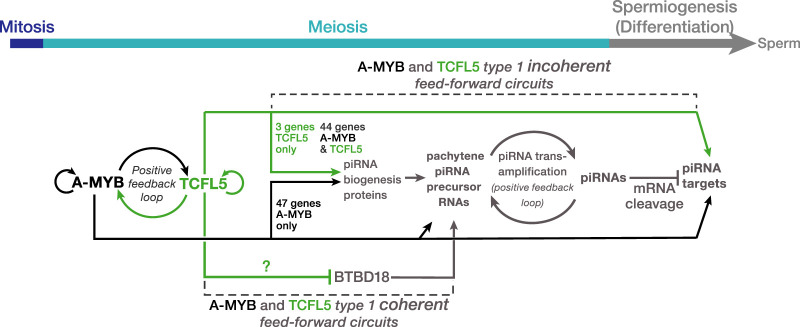
A model for the A-MYB/TCFL5 regulatory architecture of mouse male meiotic cells. The model incorporates hypotheses from Li et al. (2013), Özata et al. (2020), Wu et al. (2020), Choi et al. (2021), and this study. The figure highlights the roles of A-MYB and TCFL5 in regulating pachytene piRNA genes and direct targets of pachytene piRNAs during mouse spermatogenesis.

Our data, together with those from previous studies (Li et al. 2013; Özata et al. 2020; Wu et al. 2020; Choi et al. 2021), suggest a model for the evolutionarily conserved transcriptional architecture by which A-MYB and TCFL5 collaborate to regulate pachytene piRNA production during male meiosis I (Fig. 5). Together, A-MYB and TCFL5 directly promote transcription of piRNA biogenesis machinery and the pachytene piRNA genes, whose transcripts are the sources piRNAs. Together and separately, A-MYB and TCFL5 participate in coherent feedforward loops that promote accumulation of pachytene piRNAs.

A-MYB and TCFL5 also promote transcription of chromatin-modifying enzymes and at least one enzyme required for the biosynthesis of acyl-CoA, the acyl carrier used by the transferases that modify histone lysines to facilitate active transcription of the pachytene piRNA genes. This transcriptional architecture is present in macaque, suggesting it is conserved among placental mammals. A-MYB also regulates male meiosis in chickens (Li et al. 2013). The chicken genome encodes TCFL5, raising the possibility that the overall architecture of the A-MYB/TCFL5 regulatory circuit predates the divergence of mammals and birds.

How do new pachytene piRNA-producing loci evolve? We suggest that three events promote the emergence of new pachytene piRNA loci: direct or indirect transcriptional activation of the locus by A-MYB or TCFL5; initiation of piRNA production from the newly transcribed locus by a piRNA from an older pachytene piRNA locus; and, finally, targeting of the same or another older pachytene piRNA gene by an initiator piRNA from the novel locus. Given that the majority of mouse pachytene piRNA genes are restricted to murine rodents or mice, co-option of noncoding transcripts into the piRNA pathway seems surprisingly easy. The resulting novel piRNAs are unlikely to provide a selectable advantage to the male mouse: surprisingly few piRNAs from even the most ancient pachytene piRNA genes appear to regulate genes required for the production of functional sperm (Wu et al. 2020; Choi et al. 2021). Supporting this view, human pachytene piRNA genes are among the most rapidly diverging sequences in human genome (Özata et al. 2020). In fact, the main function of pachytene piRNAs seems to be to promote their own biogenesis. Do pachytene piRNAs really exist mainly to ensure their own production? We speculate that a small number of piRNA species modestly enhance spermatogenesis or spermiogenesis, thereby ensuring the inheritance of the tens of thousands of selfish piRNAs with no utility in germ cell development. Do most pachytene piRNAs benefit the mouse in subtle ways that remain to be discovered, or are pachytene piRNAs simply tiny narcissists that have turned the ancestral transposon silencing function of piRNAs against their host, joining the ranks of the longer selfish genetic elements present in the mammalian genome? Distinguishing between these hypotheses promises to be both challenging and rewarding, like pachytene piRNAs themselves.

## MATERIALS AND METHODS

### Mice

Mice were maintained and used according to the guidelines of the Institutional Animal Care and Use Committee of the University of Massachusetts Medical School (A201900331). C57BL/6J mice (RRID: IMSR_JAX:000664) were used as wild-type controls. *A*-*Myb*^*repro9*^ (MGI3512907) were a gift from John Schimenti, and were maintained in a C57BL/6 background.

To generate *Tcfl5*^*em1/em1*^ mutant mice, we transcribed two sgRNAs targeting sequences in *Tcfl5* intron 1 (5′-GCA GUC UGG GUA CUA GAUA G-3′) and intron 3 (5′-AUU CAC UCA AAC AAC AAG AG-3′) using T7 RNA polymerase. After purification by electrophoresis through a denaturing 10% polyacrylamide/urea gel, both sgRNAs (20 ng/µL each) and Cas9 mRNA (50 ng/µL, TriLink Biotechnologies, L-7206) were injected into the pronucleus and cytoplasm of fertilized eggs (Transgenic Animal Modeling Core, University of Massachusetts Medical School), and 15–25 blastocysts were transferred into uterus of pseudopregnant ICR females at day E2.5. Pups were screened by PCR of genomic DNA extracted from tail tissue using primers (5′-TGC CTC ATC TGG GTG GAT ATC TGA-3′; 5′-TAC ACG AAA ACA AAA CTT AAG CGG T-3′) designed to detect deletion of *Tcfl5* exons 2 and 3.

*Tcfl5*^*+/em2(flag)Pdz*^ (*Tcfl5*^*+/FLAG*^) was generated by inserting a 3XFLAG tag in-frame with TCFL5 at the endogenous *Tcfl5* locus (Cyagen). Briefly, two guide RNAs (5′-GCC GCG CCG CGC CGG CAT GTC GG-3′ and 5′-CCG CGC CGC GCC GGC ATG TCG GG-3′) targeting sequences at the 5′ end of the *Tcfl5* coding sequence, the donor vector containing the FLAG-HA-tag cassette, and Cas9 mRNA were injected into fertilized mouse eggs. F0 founders were bred to wild-type mice to identify animals with germline transmission in the F1.

### Isolation of mouse germ cells by FACS

Germ cell sorting was as described (Gainetdinov et al. 2018; Özata et al. 2020). Briefly, testis was decapsulated, incubated with 1× Gey′s Balanced Salt Solution (GBSS, Sigma, G9779) containing 0.4 mg/mL collagenase type 4 (Worthington; LS004188) at 33°C for 15 min, and the seminiferous tubules washed twice with 1× GBSS, and then incubated with 1× GBSS containing 0.5 mg/mL trypsin and 1 µg/mL DNase I at 33°C for 15 min. Next, the seminiferous tubules were maintained at 4°C on ice and gently pipetted up and down for 3 min through a Pasteur pipette to homogenize. Trypsin was inactivated with fetal bovine serum (FBS; f.c. 7.5% [v/v]), and the cell suspension was passed through a prewetted 70 µm cell strainer. Cells were recovered by centrifugation at 300*g* at 4°C for 10 min, resuspended in 1× GBSS containing 5% (v/v) FBS, 1 µg/mL DNase I, and 5 µg/mL Hoechst 33342 (Thermo Fisher, 62249), and incubated at 33°C for 45 min rotating at 150 rpm. Finally, propidium iodide (0.2 µg/mL, f.c.; Thermo Fisher, P3566) was added, and cells were filtered through a prewetted 40 µm cell strainer. Cell sorting (FACS Core, University of Massachusetts Medical School was as described [Bastos et al. 2005; Özata et al. 2020]).

### Testis histology and immunohistochemistry

Wild-type and *Tcfl5*^*em1/em1*^ mutant testis tissue was fixed with Bouin's solution, and paraffin embedded tissue sectioned at 5 µm thickness, and then stained with hematoxylin and eosin (H&E) solutions (Morphology Core Facility, University of Massachusetts Medical School). Immunohistochemistry (IHC) was performed using a standard protocol. Briefly, testis sections were deparaffinized with xylene, dehydrated with ethanol, and then boiled in 1 mM citrate buffer (pH 6.0) to retrieve antigens. To inactivate endogenous peroxidase, tissue sections were incubated with 3% (v/v) hydrogen peroxide at room temperature for 10 min, then blocked with 5% (v/v) horse serum (ImmPRESS HRP Anti-Rabbit IgG [Peroxidase] Polymer Detection Kit; Vector labs; MP-7401). Sections were incubated with rabbit anti-TCFL5 (Sigma, HPA055223; 1:500 dilution), or rabbit anti-A-MYB (Sigma, HPA008791; 1:500 dilution) antibodies at 4°C overnight. Next, secondary HRP anti-rabbit antibody (Vector labs; MP-7401) was added and incubated at room temperature for 1 h, followed by incubation with chromogenic substrate (Fisher Scientific, TA-125-QHDX). Slides were counterstained with hematoxylin and covered with a #1 coverslip. IHC images were captured using a Leica DMi8 microscope equipped with a 63×, 1.4 NA oil immersion objective.

### RNA fluorescent in situ hybridization

Paraffin embedded testes tissue was sectioned at 5 µm thickness, then deparaffinized with xylene and dehydrated with ethanol. RNAscope Hydrogen Peroxide (H_2_O_2_) (ACD, 322335) was used to inactivate endogenous peroxidase and antigens retrieved using RNAscope Target Retrieval Reagents (ACD, 322000) followed by treatment with RNAscope Protease Plus (ACD, 322331) according to the manufacturer's directions. RNAscope 2.5 HD Detection Reagents-RED assay (ACD, 322360) were used to hybridize probes for *Tcfl5* (ACD, *Tcfl5* mRNA probe, NPR-0002511, targeting 202-1589 of NM_178254.3) and *A*-*MYB* (*A*-*MYB* mRNA probe, NPR-0002510, targeting 1165–2241 of NM_008651.3) mRNAs and to amplify and detect signal. Stained slides were sealed with EcoMount (Biocare Medical, EM897L) and imaged using a Leica DMi8 microscope equipped with a 63×, 1.4 NA oil immersion objective. For simultaneous two-color detection of *Tcfl5* and *A*-*MYB* mRNAs, we used RNAscope two-color Fluorescent Detection v2 assay (ACD, 323110) and probes for *Tcfl5* (ACD, *Tcfl5* mRNA C3 probe, 815341-C3, targeting 202-1589 of NM_178254.3) and *A*-*MYB* (*A*-*MYB* mRNA C3 probe, 815331-C3, targeting 1165-2241 of NM_008651.3) mRNAs. Opal 520 reagent (Akoya Biosciences, FP1487001KT) was used to detect fluorescent signal for *Tcfl5* and Opal 690 reagent (Akoya Biosciences, FP1497001KT) for *A*-*Myb*. DAPI (1 mg/ml; Thermo Fisher Scientific, 62248) was used to counterstain nuclei. Stained slides were sealed using gold antifade mounting solution (Thermo Fisher Scientific, P36930), covered with a #1 coverslip, and *Z*-stack images captured using a Leica SP8 Lightning Confocal Microscope equipped with a 63×, 1.4 NA oil immersion objective.

### Western blotting

Frozen testis tissues were homogenized in a Dounce homogenizer using 20 strokes of pestle B in RIPA lysis buffer (25 mM Tris-HCl, pH 7.6, 150 mM NaCl, 1% [v/v] NP-40, 1% sodium deoxycholate, and 0.1% [w/v] SDS) containing 1× homemade protease inhibitor cocktail [1 mM 4-(2-Aminoethyl)benzenesulfonyl fluoride hydrochloride (Sigma; A8456), 0.3 µm Aprotinin, 40 µm Betanin hydrochloride, 10 µm. E-64 (Sigma; E3132), 10 µm Leupeptin hemisulfate]. Lysed tissue was then sonicated (Branson Digital Sonifier; 450 Cell Disruptor) to break nuclei. After sonication, the samples were centrifuged at 20,000*g* at 4°C for 30 min. The supernatant was transferred to a new tube, and protein concentration measured using the Pierce BCA Protein Assay Kit (Thermo Fisher; 23225). Total protein (50 µg) from each sample was mixed with 1/4 volume of loading dye (106 mM Tris-HCl, pH 6.8, 141 mM Tris base, 2% SDS, 10% v/v glycerol, 0.51 mM EDTA, 0.22 mM SERVA Blue G, and 0.175 mM Phenol Red) containing 0.2 M dithiothreitol and heated at 95°C for 6 min to denature proteins. Samples were resolved by electrophoresis through a 4%–20% polyacrylamide gradient SDS gel (Thermo Fisher, XP04205BOX), and the gel transferred to PVDF membrane (Millipore, IPVH00010). The membrane was blocked with Blocking Buffer (Rockland Immunochemicals, MB-070) at room temperature for 1.5 h and incubated with rabbit polyclonal anti-A-MYB (Sigma, HPA008791; 1:1000 dilution), rabbit polyclonal anti-TCFL5 (Abgent, AP17006b; 1:1000 dilution), or mouse monoclonal anti-FLAG (Sigma, F3165; 1:1000) antibody at 4°C overnight. Next, the membrane was washed three times (30 min each) with 1× PBS-T (0.1% [v/v] Tween-20 in 1× PBS) at room temperature; incubated with secondary donkey anti-rabbit IRDye 680RD antibody (LI-COR, 926-68073; 1:15,000 dilution) at room temperature for 30 min; washed three times (30 min each) with 1× PBS-T at room temperature, and signal detected using the Odyssey Infrared Imaging System (LI-COR). As a loading control, membrane was incubated with mouse anti-ACTIN antibody (Santa Cruz Biotechnology, sc-47778; 1:10,000 dilution) at room temperature for 2 h, followed by goat anti-mouse IRDye 800CW secondary antibody (LI-COR, 926-32210; 1:15,000 dilution) as described above.

### Small-RNA library construction and analysis

Total RNA was extracted from frozen testis tissue using the mirVana miRNA Isolation Kit (Thermo Fisher, AM1560). Small RNA library construction was performed as described (Gainetdinov et al. 2018). Briefly, total RNA was resolved by electrophoresis through a denaturing 15% polyacrylamide gel to select 18–34 nt RNAs. Size-selected small RNA was oxidized with sodium periodate (NaIO_4_) prior to 3′ adaptor ligation to suppress ligation to microRNAs and siRNAs. The 3′ adaptor (5′-rApp NNN TGG AAT TCT CGG GTG CCA AGG /ddC/-3′) was ligated in the presence of 50% (w/v) PEG8000 (Rigaku, 1008063). The 3′ adaptor ligated small RNAs were then purified by electrophoresis through a denaturing 15% polyacrylamide gel to eliminate the unligated 3′ adaptor; 42 to 60 nt small RNA containing the 3′ adaptor were recovered from the gel. Finally, 5′ adaptor was ligated to the RNA and cDNA generated. Libraries were sequenced (79 nt single-end reads) using a NextSeq500 (Illumina).

After removing 3′ adaptor sequence from the reads, we eliminated reads with PHRED score <5. Remaining reads were then mapped to mouse genome (mm10) using piPipes (Han et al. 2015), using Bowtie 2.2.5 (Langmead and Salzberg 2012), allowing one mismatch. We quantified the length distribution and abundance of piRNA reads by normalizing to genome-mapping reads reported as reads per million (rpm). Spike-in oligonucleotides were identified allowing no mismatches. The absolute quantity of piRNAs was calculated based on the read abundance of the spike-in RNAs. Small RNA sequencing statistics are provided in Supplemental Table S4.

### Long RNA library construction and analysis

Total RNA from frozen testis samples and from sorted germ cells was extracted using mirVana miRNA Isolation Kit (Thermo Fisher, AM1560). Before RNA library construction from FACS-purified germ cells, we added 1 µl of 1:100 dilution of ERCC spike-in mix 1 (Thermo Fisher, 4456740, LOT00418382) to 0.5–1 µg total RNA to enable subsequent molecular quantification of transcripts. Library construction was performed as described (Zhang et al. 2012) with a modified ribosomal RNA (rRNA) removal procedure. Briefly, 186 short antisense DNA oligos (ASOs; 50–80 nt) complementary to mouse rRNAs were designed and pooled (0.05 µM/each) (Morlan et al. 2012; Adiconis et al. 2013). Pooled rRNA ASO (1 µL) were added to 1 µg total RNA in the presence of 100 mM Tris-HCl (pH 7.4) and 200 mM NaCl, heated to 95°C and cooled to 22°C at −0.1°C/s, then incubated at 22°C for 5 min. The DNA:RNA hybrids were digested with 10 U Thermostable RNase H (Epicentre, H39500) in 50 mM Tris-HCl (pH 7.4), 0.1 M NaCl, and 20 mM MgCl_2_ at 45°C for 30 min. Next, samples were treated with 4 U Turbo DNase (Thermo Fisher, AM2238) at 37°C for 20 min. To enrich RNA >200 nt and remove tRNA, RNA samples were purified with an RNA Clean & Concentrator-5 (Zymo Research, R1015), and cDNA generated as described. Libraries were sequenced as 79 + 79 nt paired-end reads using a NextSeq500 (Illumina).

rRNA reads were eliminated using Bowtie 2.2.5 with default parameters (Langmead and Salzberg 2012), and then the remaining reads were mapped to the mouse genome (mm10) using STAR 2.3 (Dobin et al. 2013). Results in SAM format were deduplicated and transformed into BAM format using SAMtools 1.8 and Umitools (Li et al. 2009; Fu et al. 2018). Finally, mapped reads were assigned to protein-coding genes, long noncoding RNAs (lncRNAs), and piRNA genes using HTSeq 0.9.1 (Anders et al. 2015). Transcript abundance was reported as reads per million uniquely mapped reads per thousand nucleotides (RPKM) calculated using homemade Bash scripts.

Transcript abundance in molecules per cell was performed as described (Gainetdinov et al. 2018). Because each sample contained ∼623,291,645 molecules of ERCC spike-in mix, the abundance of each gene = (number of mapped reads × 623,291,645)/(number of cells used to prepare the library × the number of reads mapping to the ERCC spike-in sequences). RNA sequencing statistics are provided in Supplemental Table S4. All analyses used Ensembl-v86 gene annotations.

### Chromatin immunoprecipitation and sequencing

Chromatin immunoprecipitation (ChIP) was performed as described (Li et al. 2013; Özata et al. 2020). Briefly, frozen testis tissue was minced on dry ice and incubated in 1.7 mL tubes in ice-cold PBS containing 2% (w/v) formaldehyde at room temperature for 30 min, slowly tumbling end-over-end. Fixed tissue was crushed in ChIP lysis buffer (1% SDS, 10 mM EDTA, 50 mM Tris-HCl, pH 8.1) with a Dounce homogenizer using 40 strokes of pestle B (Kimble-Chase). To shear chromatin to 150–200 bp, samples were sonicated (Covaris, E220). Sonicated lysate was then diluted 1:10 with ChIP dilution buffer (0.01% SDS, 1.1% Triton X-100, 1.2 mM EDTA, 16.7 mM Tris-HCl, pH 8.1, 167 mM NaCl) and immunoprecipitated using 5 µg anti-TCFL5 (Sigma, HPA055223, lot # 000000907) or 5.5 µg anti-A-MYB (Sigma, HPA008791, lot # C105987) antibody. After immunoprecipitation, DNA was extracted with phenol:chloroform:isoamyl alcohol (25:24:1; pH 8), and ChIP-seq libraries generated as described (Zhang et al. 2012). Libraries were sequenced as 79 + 79 nt paired-end reads using a NextSeq500 (Illumina).

Raw reads were mapped to the mouse genome (mm10) using Bowtie 2.2.5 with parameters -very-sensitive -no-unal -no-mixed -no-discordant -|10. Duplicate reads were removed using SAMtools 1.8 (Li et al. 2009). Normalized genome coverage files in bigWig format were generated using a homemade script. Data are reported as reads per million (rpm) using uniquely mapping reads normalized to read depth. Significant TCFL5 or A-MYB peaks (FDR < 0.05 for enrichment relative to input) were identified using MACS 1.1.2 (Zhang et al. 2008) with parameters -keep-dup all -q 0.001. ChIP sequencing statistics are provided in Supplemental Table S4.

The distance (bp) of transcription start site to the nearest TCFL5 or A-MYB peak summit for each gene was calculated. Genes that retain TCFL5 or A-MYB peak within the range of 500 bp around their transcription start site were considered as TCFL5- or A-MYB-regulated genes.

### CUT&RUN sequencing

Cleavage under targets & release using nuclease (CUT&RUN) sequencing from FACS-purified germ cells was performed as described (Skene and Henikoff 2017). Briefly, FACS-purified germ cells were lysed using nuclear extraction buffer (20 mM HEPES-KOH, pH 7.9, 10 mM KCl, 0.5 mM spermidine, 0.1% Triton X-100, 20% glycerol) and nuclei recovered by centrifugation at 600*g* at 4°C for 3 min. Nuclei were immobilized on BioMag Plus Concanavalin A-coated paramagnetic beads (Bangs Laboratories, BP531) at room temperature for 20 min. After immobilizing on beads, nuclei were blocked in 20 mM HEPES-KOH, pH 7.5, 150 mM NaCl, 0.5 mM spermidine, 0.1% BSA, 2 mM EDTA) at room temperature for 5 min. Then, 2.5 µg anti-TCFL5 (Sigma, HPA055223 or Sigma, HPA055223, lot # 000000907) or 2.5 µg anti-A-MYB (Sigma, HPA008791, lot # C105987) antibodies were added to the beads and incubated at 4°C for 4 h, slowly tumbling end-over-end. CUT&RUN without antibody provided a negative control. After antibody incubation, beads were washed twice in wash buffer (20 mM HEPES-KOH, pH 7.5, 150 mM NaCl, 0.5 mM spermidine, 0.1% BSA) and incubated with 1.5 µL protein A-MNase fusion protein (pA-MN; 140 µg/mL) at 4°C for 2 h, slowly tumbling end-over-end. To remove unbound pA-MN, beads were washed twice in wash buffer. To liberate DNA bound by TCFL5 or A-MYB, 3 µL 100mM CaCl_2_ was added to the beads and incubated at 0°C for 7 min. The reaction was stopped by adding an equal volume of 2× STOP buffer (200 mM NaCl, 20 mM EDTA, 4 mM EGTA, 50 µg/mL RNase A, 40 µg/mL glycogen) and then incubated at 37°C for 20 min. Nuclei were removed by centrifugation at 16,000*g* at 4°C. The supernatant containing CUT&RUN fragments was transferred to a fresh 1.7 mL tube. After extracting the DNA with phenol:chloroform:isoamyl alcohol (25:24:1; pH 8), CUT&RUN libraries were prepared as described (Zhang et al. 2012) with slight modifications. Briefly, end-repair using 0.5 U/µL T4 DNA polymerase (NEB, M0203), and adenylation using 0.5 U/µL *Taq* DNA polymerase (NEB, M0273), were carried out in a single reaction. Then, end-repaired and adenylated DNA was purified using AMPure beads. Finally, Illumina adapters (MP-Ada1: 5′-p GAT CGG AAG AGC ACA CGT CT-3′; MP-Ada2: 5′-p ACA CTC TTT CCC TAC ACG ACG CTC TTC CGA TCT-3′) were added using 600 U/µL T4 DNA ligase (Enzymatics Inc., L603-HC-L) at room temperature for 30 min.

CUT&RUN fragments were sequenced using 79 + 79 nt paired-end reads on a NextSeq500 (Illumina). Raw reads were mapped to the mouse genome (mm10) using Bowtie 2.2.5 (Langmead and Salzberg 2012) with parameters -very-sensitive -no-unal -no-mixed -no-discordant -|10. TCFL5 and A-MYB peaks were identified used Sparse Enrichment Analysis for CUT&RUN (SEARC [Meers et al. 2019]) in “norm relaxed” mode. CUT&RUN performed without antibody was used as the background control. Genes with a TCFL5 or A-MYB peak ±500 bp of their transcription start site were considered as TCFL5- or A-MYB-regulated. To be considered bound by TCFL5, a gene was required (i) to have a SEARC-identified peak within 500 bp of its transcription start site in both CUT&RUN replicates; or (ii) to have a SEARC-identified peak within 500 bp of its transcription start site in one of the two CUT&RUN replicates and a MACS2-identified peak within 500 bp of its transcription start site in two of three ChIP-seq replicates. To be considered bound by A-MYB, a gene was required (i) to have a SEARC-identified peak within 500 bp of its transcription start site in both CUT&RUN replicates; or (ii) to have a SEARC-identified peak within 500 bp of its transcription start site in one of the two CUT&RUN replicates and a MACS2-identified peak within 500 bp of its transcription start site in ChIP-seq. CUT&RUN sequencing statistics are provided in Supplemental Table S4.

### Statistics

All statistics were calculated using R console (https://www.rstudio.com); graphs were generated using R console or Prism 8.4.3 (GraphPad Software, LLC). For box plots, boxes represent the first and third quartiles. Outliers are defined as data points with values >third quartile +1.5 × IQR or lower than the first quartile −1.5 × IQR, where IQR (interquartile range) is the maximum third quartile minus the first quartile minimum. Two-sided Mann–Whitney–Wilcoxon U and Fisher's exact tests were used to calculate *P*-values.

## DATA DEPOSITION

Sequencing data are available from the National Center for Biotechnology Information Sequence Read Archive using Gene Expression Omnibus accession number GSE166104. Code used to identify putative piRNA-directed cleavage sites is available at https://github.com/weng-lab/GTBuster.

## SUPPLEMENTAL MATERIAL

Supplemental material is available for this article.

## Supplementary Material

Supplemental Material
