# Supplementary material for: A-MYB/TCFL5 regulatory architecture ensures the production of pachytene piRNAs in placental mammals
Source: RNA. 2023 Jan;29(1):30–43. doi: 10.1261/rna.079472.122 (PMC9808571; doi:10.1261/rna.079472.122)
Supplement: Supplemental Material [file supp_29_1_30__DC1.html]

A-MYB/TCFL5 regulatory architecture ensures the production of pachytene piRNAs in placental mammals — A-MYB/TCFL5 regulatory architecture ensures the production of pachytene piRNAs in placental mammals — Supplemental Material 

# A-MYB/TCFL5 regulatory architecture ensures the production of pachytene piRNAs in placental mammals

## Supplemental Material

- Supplemental\_Figure\_S1.pdf
- Supplemental\_Figure\_S2.pdf
- Supplemental\_Figure\_S3.pdf
- Supplemental\_Figure\_S4.pdf
- Supplemental\_Figure\_S5.pdf
- Supplemental\_Legends.pdf
- Supplemental\_Table\_S1.xlsx
- Supplemental\_Table\_S2.xlsx
- Supplemental\_Table\_S3.xlsx
- Supplemental\_Table\_S4.xlsx
