# Supplementary figures and images for: A-MYB/TCFL5 regulatory architecture ensures the production of pachytene piRNAs in placental mammals
Source: RNA. 2023 Jan;29(1):30–43. doi: 10.1261/rna.079472.122 (PMC9808571; doi:10.1261/rna.079472.122)

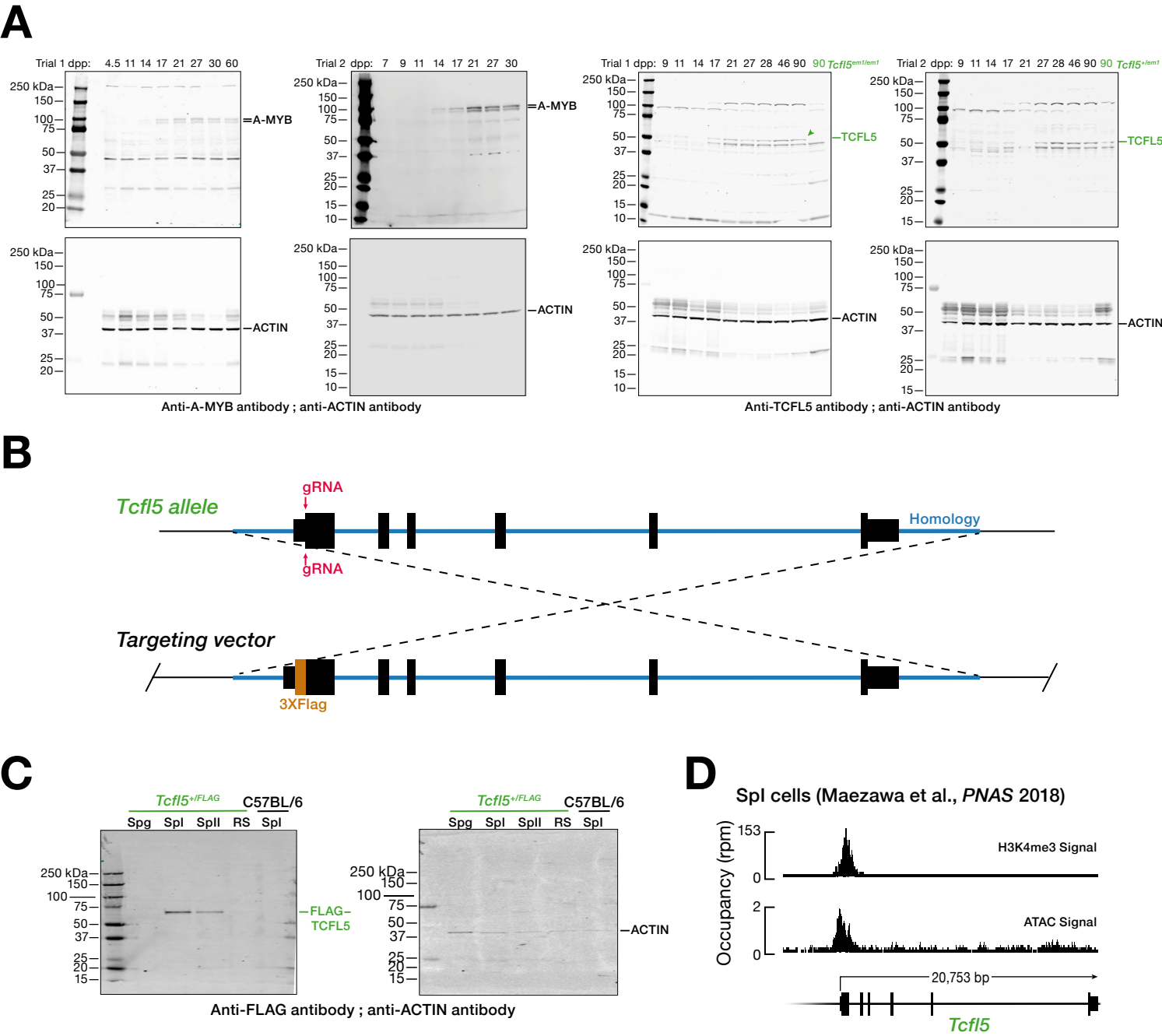

Supplement: Supplemental Material [file supp_079472.122_Supplemental_Figure_S1.pdf]

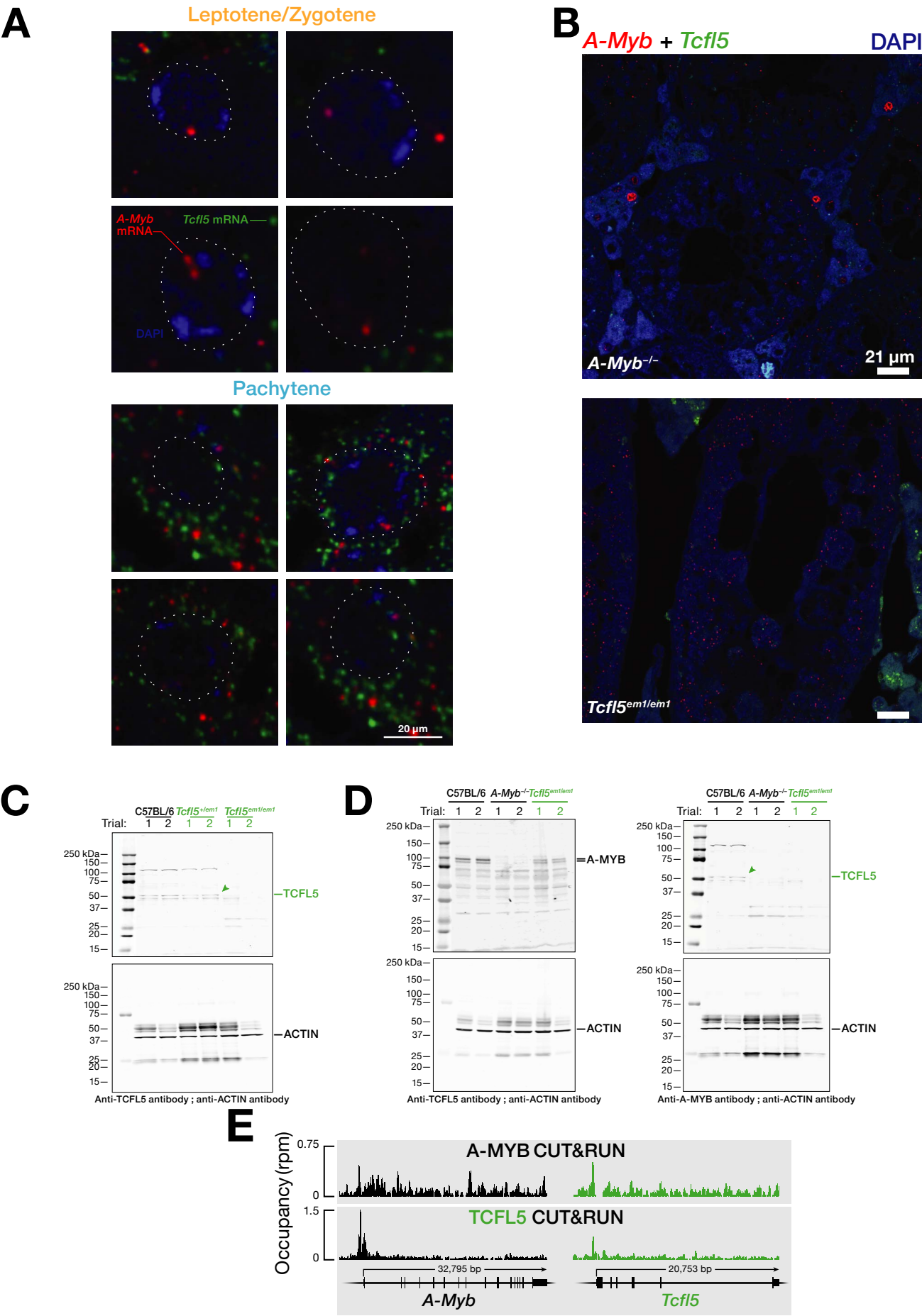

Supplement: Supplemental Material [file supp_079472.122_Supplemental_Figure_S2.pdf]

**A**

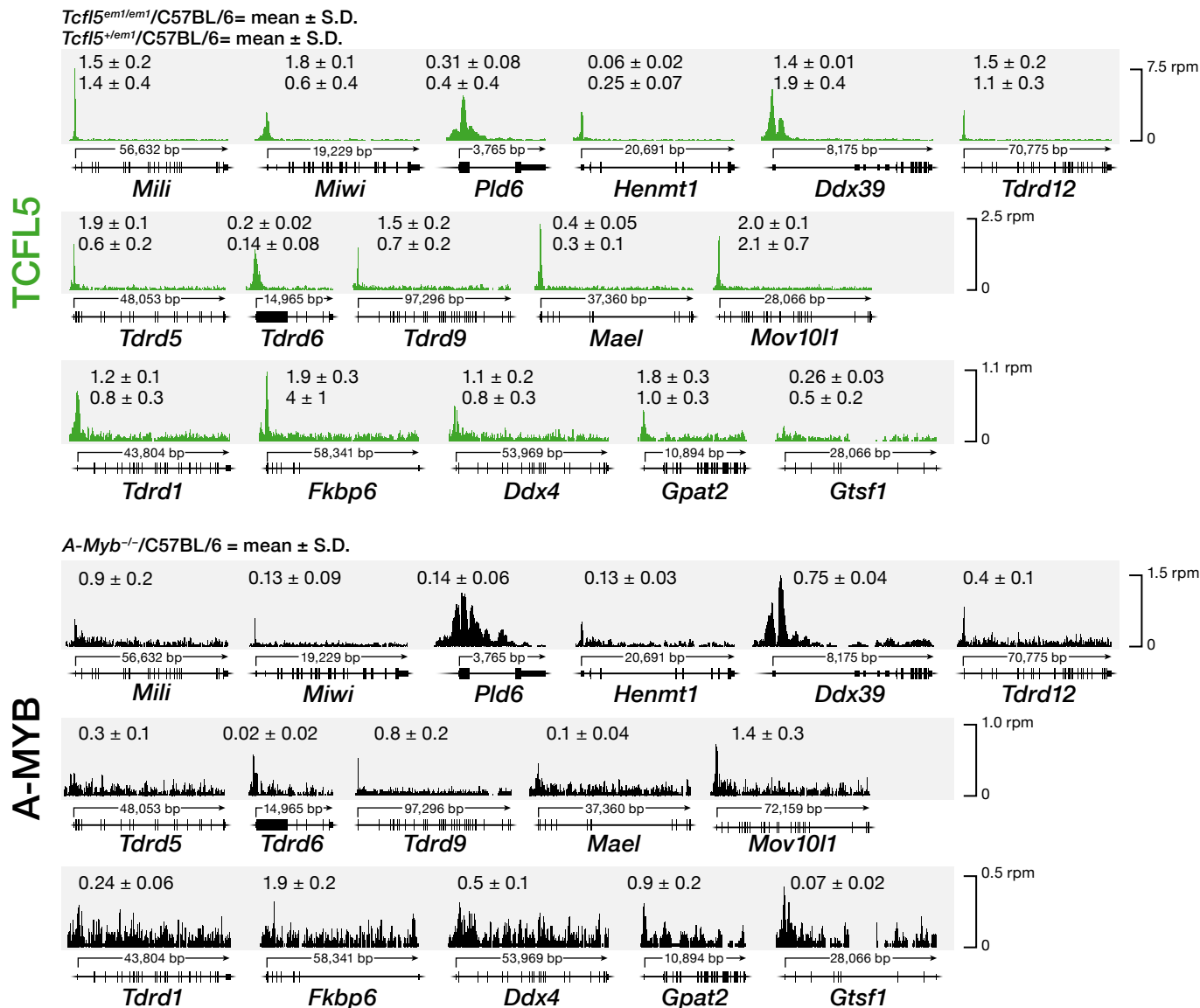

**B**

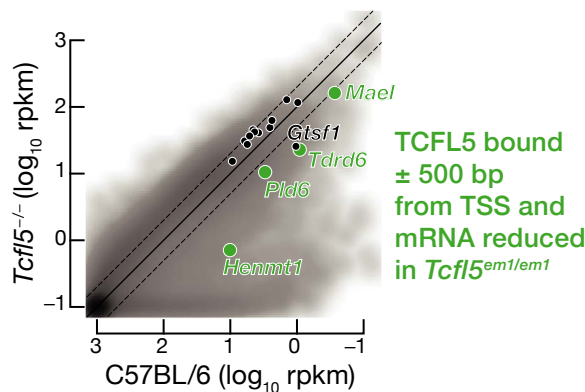

Supplement: Supplemental Material [file supp_079472.122_Supplemental_Figure_S3.pdf]

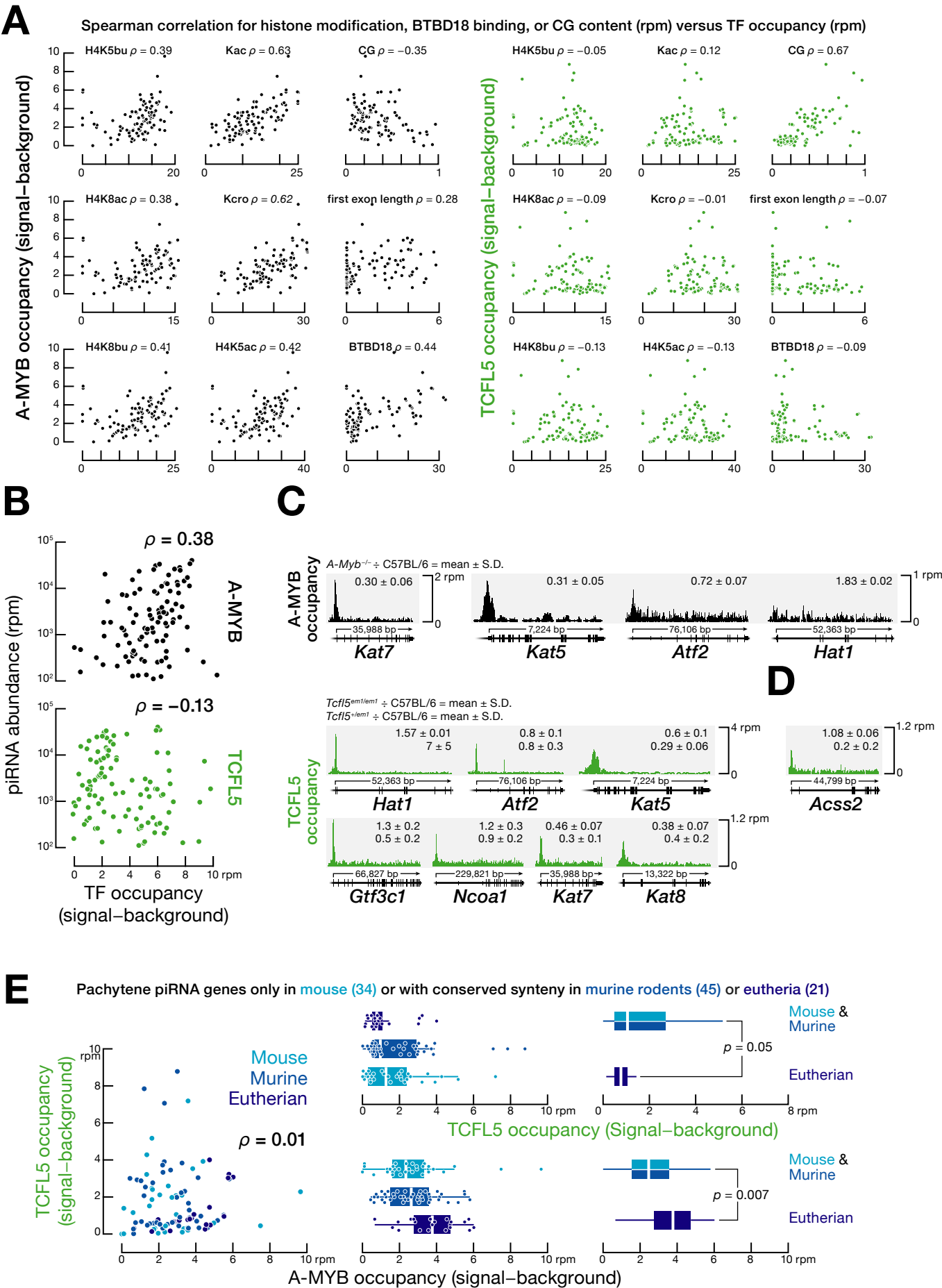

Supplement: Supplemental Material [file supp_079472.122_Supplemental_Figure_S4.pdf]

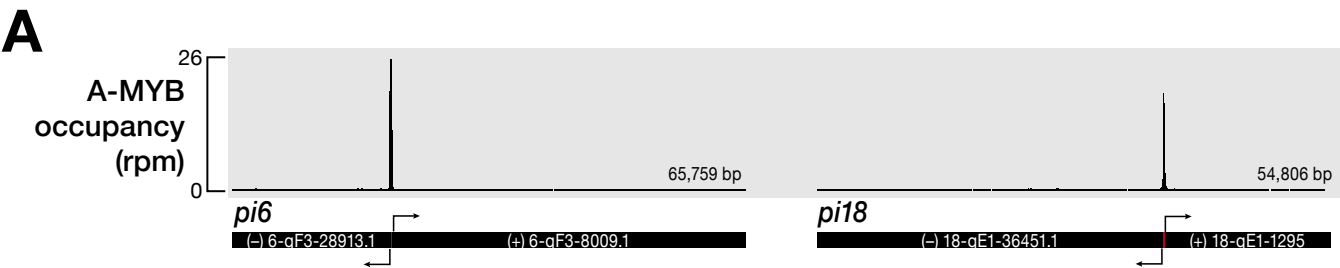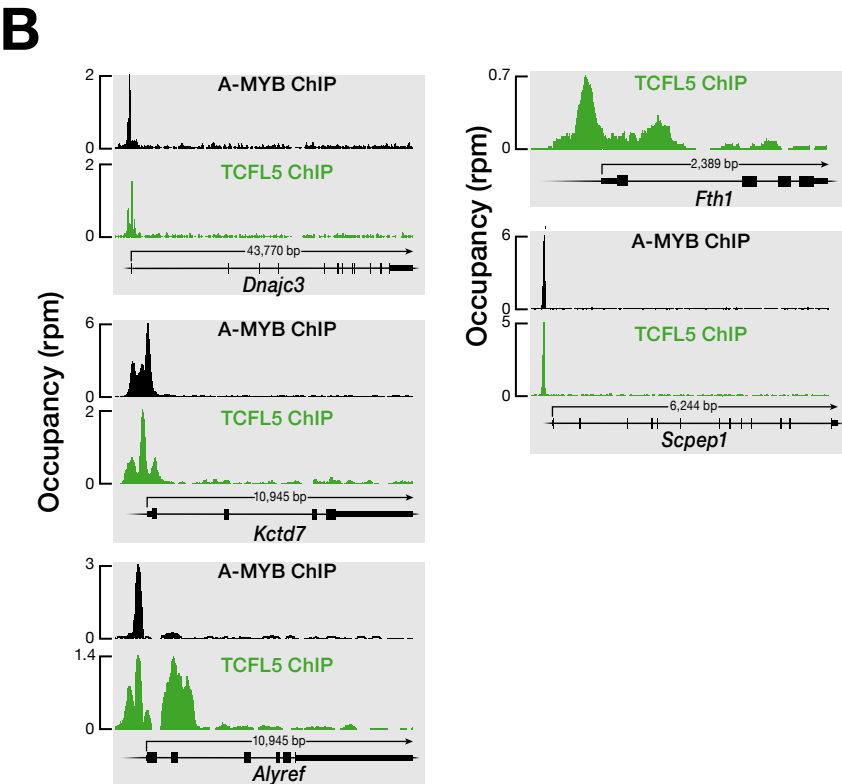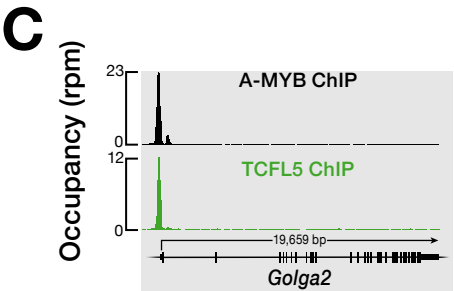

Supplement: Supplemental Material [file supp_079472.122_Supplemental_Figure_S5.pdf]
