# Supplementary material for: A-MYB/TCFL5 regulatory architecture ensures the production of pachytene piRNAs in placental mammals
Source: RNA. 2023 Jan;29(1):30–43. doi: 10.1261/rna.079472.122 (PMC9808571; doi:10.1261/rna.079472.122)
Supplement: Supplemental Material [file supp_079472.122_Supplemental_Legends.pdf]

Supplementary Materials for

A-MYB/TCFL5 regulatory architecture ensures the production of pachytene piRNAs in placental mammals

Adriano Biasini, Tianxiong Yu, Katharina Cecchini, Martin Säflund, Haiwei Mou, Amena Arif, Atiyeh Eghbali, Dirk G. de Rooij, Zhiping Weng, Phillip D. Zamore,* and Deniz M. Özata*

*Corresponding author. Email: phillip.zamore@umassmed.edu and deniz.ozata@su.se

Supplemental Fig. S1.

Temporal and spatial expression of A-MYB and TCFL5.

(A) Protein abundance of A-MYB and TCFL5 in the testis of staged mouse. (50 µg testis protein per lane). ACTIN serves as a loading control.

(B) Strategy for creating knock-in mice whose endogenous TCFL5 protein is tagged with 3XFLAG peptide at its amino-terminus ($Tcfl5^{+/FLAG}$).

(C) FACS-purified germ cells from $Tcfl5^{+/FLAG}$ mice (protein from ~75,000 germ cells per lane)

(**D**) H3K4me3 and ATAC signals round the transcription start site of *Tcfl5* gene (Maezawa et al., 2018).

Supplemental Fig. S2.

Reciprocal positive feedback loops between A-MYB and TCFL5.

(A) Representative two-color RNA-FISH images of nuclei from individual leptotene/zygotene and pachytene cells from **Fig. 2A**.

(B) *A-Myb* and *Tcfl5* mRNAs were detected in the seminiferous tubules of *Tcfl5*$^{em1/em1}$ and *A-Myb*$^{-/-}$ mutant mice using two-color RNA-FISH.

(C) Abundance of TCFL5 protein in *Tcfl5*$^{em1/em1}$ and *Tcfl5*$^{+/em1}$ mutant testes, compared to C57BL/6 wild-type, was measured by immunoblotting. ACTIN serves as a loading control. Each lane contained 50 µg testis protein.

(D) Protein abundance of A-MYB and TCFL5 from *A-Myb*$^{-/-}$ and *Tcfl5*$^{em1/em1}$ mutant mice testes was measured by immunoblotting. ACTIN serves as a loading control. Each lane contained 50 µg testis protein.

(E) A-MYB and TCFL5 occupancy at the promoters of the *A-Myb* and *Tcfl5* genes was measured using CUT&RUN.

Supplemental Fig. S3.

TCFL5 drives the transcription of mRNAs encoding mouse piRNA biogenesis proteins.

(A) A-MYB and TCFL5 CUT&RUN peaks at the promoters of 16 genes that encode piRNA biogenesis proteins.

(B) Scatter plot of the steady-state mRNA abundance of piRNA biogenesis genes in *Tcfl5*$^{em1/em1}$ mutant mice.

Supplemental Fig. S4.

TCFL5 regulates evolutionarily younger, pachytene piRNA-producing genes.

(A) Spearman correlation (ρ) between A-MYB or TCFL5 occupancy and the evolutionarily conserved features of pachytene piRNA genes (i.e., histone modification, BTBD18 occupancy, CG content, and first exon length).

(B) Spearman correlation between A-MYB or TCFL5 occupancy and piRNA abundance.

(C) A-MYB and TCFL5 CUT&RUN peaks at the promoters of genes encoding histone acylation enzymes: *Hat1*, *Atf2*, *Kat5*, *Kat6a*, *Gtf3c1*, *Ncoa1*, *Kat7*, and *Kat8*. The change in steady-state mRNA expression in *Tcfl5*$^{em1/em1}$ whole testes and *Tcfl5*$^{+/em1}$ mutant primary spermatocytes, relative to C57BL/6 controls, is reported for each gene as mean ± SD.

(D) TCFL5 CUT&RUN peak at the promoter of *Acss2* gene encoding acyl-CoA synthetase.

(E) Left panel, scatter plot to test the Spearman correlation between A-MYB and TCFL5 occupancy around the TSS of pachytene piRNA genes classified by conservation of their genomic locations (synteny) among eight placental mammals. Center panels, A-MYB and TCFL5 occupancy, determined using CUT&RUN, at the promoters of the same three class of pachytene piRNA genes. Vertical lines: median; whiskers: maximum and minimum values excluding outliers (i.e., 1.5 × IQR). Each dot represents an individual pachytene piRNA gene. Right panels, same as in the center panels, except comparing A-MYB and TCFL5 occupancy for the promoters of pachytene piRNA genes with or without conserved synteny among eutherian mammals. *p*-value was calculated using a two-sided Mann-Whitney-Wilcoxon U test.

Supplemental Fig. S5.

A-MYB/TCFL5 regulatory architecture sits atop of pachytene piRNA-driven gene regulation.

(A) A-MYB CUT&RUN peak at the promoters of *pi6* and *pi18* pachytene piRNA genes.

(B) A-MYB and TCFL5 occupancies around the promoters of genes whose mRNA products are targeted by *pi6* pachytene piRNAs: *Dnajc3*, *Kctd7*, *Alyref*, *Fth1*, and *Scpep1*.

(C) TCFL5 peak around the transcription start site of *Golga2* gene whose mRNA product is targeted by *pi18* pachytene piRNAs.

Supplemental Table S1

Regulation of pachytene piRNA genes and genes encoding piRNA maturation proteins by A-MYB–TCFL5 axis.

(A) A-MYB and TCFL5 occupancy around the promoters of piRNA maturation genes. Steady-state piRNA precursor transcript abundance (RPKM) are reported.

(B) A-MYB and TCFL5 occupancy around the promoters of pachytene piRNA genes. Steady-state piRNA precursor transcript abundance (RPKM) and piRNA abundance (RPM) are reported.

Supplemental Table S2.

Reciprocal cleavage events between piRNAs and piRNA precursor transcripts from pachytene piRNA genes.

Supplemental Table S3.

List of genes regulated by TCFL5 in macaque.

Supplemental Table S4.

High-throughput sequencing statistics.
